# Investigating ultrasound–light interaction in scattering media

**DOI:** 10.1117/1.JBO.25.2.025002

**Published:** 2020-02-26

**Authors:** Yujia Huang, Michelle Cua, Joshua Brake, Yan Liu, Changhuei Yang

**Affiliations:** California Institute of Technology, Department of Electrical Engineering, Pasadena, California, United States

**Keywords:** acousto-optics, scattering, tomography

## Abstract

**Significance:** Ultrasound-assisted optical imaging techniques, such as ultrasound-modulated optical tomography, allow for imaging deep inside scattering media. In these modalities, a fraction of the photons passing through the ultrasound beam is modulated. The efficiency by which the photons are converted is typically referred to as the ultrasound modulation’s “tagging efficiency.” Interestingly, this efficiency has been defined in varied and discrepant fashion throughout the scientific literature.

**Aim:** The aim of this study is the ultrasound tagging efficiency in a manner consistent with its definition and experimentally verify the contributive (or noncontributive) relationship between the mechanisms involved in the ultrasound optical modulation process.

**Approach:** We adopt a general description of the tagging efficiency as the fraction of photons traversing an ultrasound beam that is frequency shifted (inclusion of all frequency-shifted components). We then systematically studied the impact of ultrasound pressure and frequency on the tagging efficiency through a balanced detection measurement system that measured the power of each order of the ultrasound tagged light, as well as the power of the unmodulated light component.

**Results:** Through our experiments, we showed that the tagging efficiency can reach 70% in a scattering phantom with a scattering anisotropy of 0.9 and a scattering coefficient of $4\;{\mathrm{mm}}^{-1}$ for a 1-MHz ultrasound with a relatively low (and biomedically acceptable) peak pressure of 0.47 MPa. Furthermore, we experimentally confirmed that the two ultrasound-induced light modulation mechanisms, particle displacement and refractive index change, act in opposition to each other.

**Conclusion:** Tagging efficiency was quantified via simulation and experiments. These findings reveal avenues of investigation that may help improve ultrasound-assisted optical imaging techniques.

## 1 Introduction

Light is widely used in biomedical imaging because it is nonionizing and considered safe compared to other imaging modalities, such as x-ray computed tomography.^1^ The optical properties of targeted contrast agents provide much information about biological tissue for functional imaging.^2^^–^^4^ However, one longstanding challenge in optical imaging is scattering.^5^ Due to the turbid nature of biological tissue, light will be scattered; this scattering prohibits high-resolution imaging deep in tissue.

Unlike light, ultrasound is much less scattered in biological tissue and is able to form a focused spot inside scattering media.^6^ Some experimental imaging techniques use ultrasound to help with the issue of light scattering. For instance, in ultrasound-modulated optical tomography (UOT), a fraction of the photons passing through the ultrasound beam are modulated, or “tagged.” Selective detection of these tagged photons gives rise to improved resolution.^7^^–^^11^ In wavefront-shaping-related techniques, researchers correct optical wavefront distortions measured using an approximate point source (guidestar). Ultrasound guidestars have been favored because they are noninvasive and freely movable.^12^ For example, time-reversed ultrasonically encoded (TRUE) techniques combine ultrasound modulation and optical phase conjugation to focus light inside scattering media.^13^^–^^15^ All of these techniques are based on the fact that light is tagged by ultrasound so that one can selectively detect only the light coming from the ultrasound focal spot. However, the detection of tagged light has always been a demanding task because of the small amount of tagged photons.^11^^,^^16^ Therefore, a better understanding of the interaction between ultrasound and light and quantification of tagging efficiency is crucial for estimating system signal-to-noise ratio (SNR) and designing detection methods to improve SNR in ultrasound-assisted optical imaging techniques.

One focus of this paper is to study the ultrasound-tagging efficiency in a manner consistent with its definition: the fraction of light tagged or frequency modulated (includes all frequency-shifted components), or alternatively (1, the fraction of light untagged) with respect to the light passing through the ultrasonic field. Most prior experiments on ultrasound-modulated light typically quantified the strength of the first order of frequency-shifted light or the ratio between the first-order and zeroth-order (untagged light).^7^^,^^17^^–^^19^ A recent work characterized the strength of the first order of ultrasonic-tagged light using slow light filter detection by diffusion theory calculations and experimental measurements.^20^ These approaches underestimate the actual tagging efficiency as they do not account for the tagged light that is frequency modulated at higher orders. Some approaches relying on photorefractive effects detect all the tagged light by the change of untagged light.^21^^,^^22^ However, tagging efficiency is not determined because the total amount of light is not restricted within the ultrasonic region. To better account for the amount of tagged light in practical applications, we believe it is worth studying the full ultrasound-tagging efficiency within ultrasonic field.

The second focus of this paper is to experimentally verify the contributive (or noncontributive) relationship between the mechanisms involved in the ultrasound optical modulation process. Prior works have identified three such mechanisms:^23^^,^^24^ refractive index modulation, ultrasound-induced particle displacement, and optical intensity modulation. When ultrasound propagates, the medium is compressed and rarified, resulting in refractive index changes. The changes in refractive index modulate the optical path length (OPL) of light, causing phase variations. In addition, in scattering media, ultrasound induces particle displacement, which modulates the physical path length, and thereby, the phase of the light. Both of these mechanisms impart variations in the optical phase, which causes the light frequency to be shifted by n times the ultrasound frequency, where n is the order. The zeroth-order corresponds to the untagged light. The propagation of ultrasound in the medium also leads to variations in the optical properties, such as the absorption coefficient and the scattering coefficient, causing variation in the intensity of light. This incoherent mechanism is much weaker than the first two coherent modulation mechanisms mentioned above and is typically ignored when modeling ultrasound–light interaction.^23^

Monte Carlo simulations that considered the two coherent modulation mechanisms independently have been implemented to verify the analytical model and compare the contributions from different mechanisms.^24^ However, the contributions from the two modulation mechanisms are not independent if the particles move with the background fluid,^25^ a subtlety that can significantly impact the extent of the ultrasonic modulation. The numerical simulations and experimental measurements of the aggregate contributions of these two effects are a major focus of this project. Some previous work on UOT imaging with speckle contrast change^16^^,^^26^^,^^27^ also mentioned this “anticorrelation” effect between the two mechanisms, and their experimental results on image contrast matched their Monte Carlo simulation results taking this effect into account. However, these prior works did not systematically study this effect in the context of the tagging efficiency; our work was aimed at specifically studying this effect on the tagging efficiency and provide a straightforward verification of the effect’s impact.

In this work, we used a Monte Carlo method to simulate the interaction between the two coherent modulation mechanisms to determine the overall tagging efficiency. The codes for these simulations are provided in the GitHub repository available at: https://github.com/yjhuangcd/ultrasound-tagging. The simulation results were then compared to our experimental results.

## 2 Theory

**Fig. 1:**
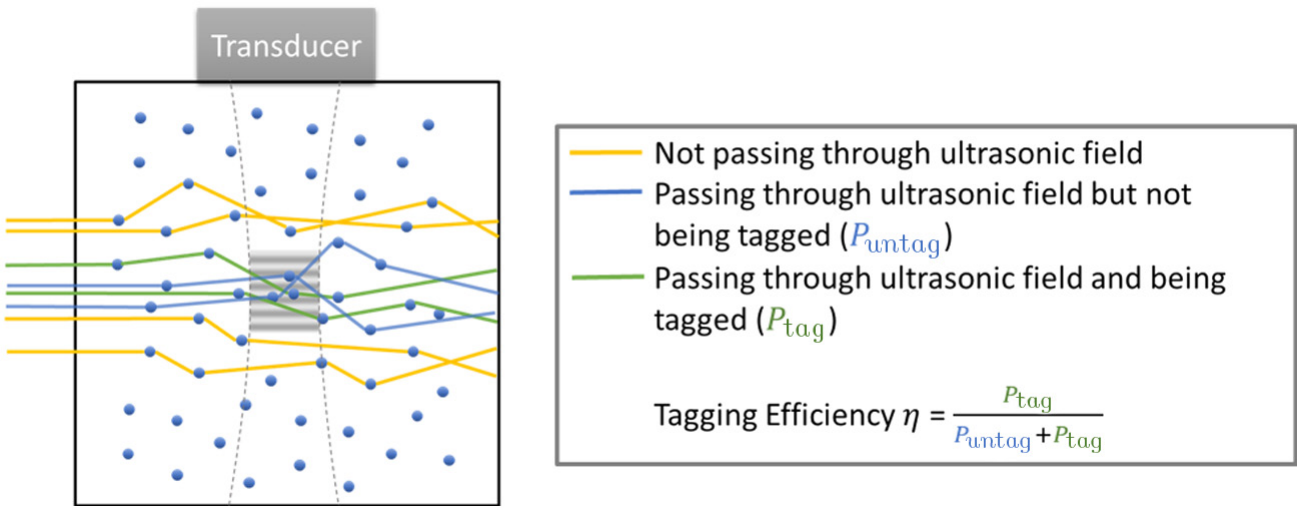
A cartoon illustration of tagging efficiency. Only light passing through the ultrasound field is considered. Tagged light refers to the light whose frequency is shifted by ultrasonic modulation.

To investigate the tagging efficiency in scattering media, we considered three types of light: (1) light that passes through the ultrasound field and is tagged by it, (2) light that passes through the ultrasound field but is not tagged by it, and (3) light that does not pass through the ultrasound field (Fig. 1). The tagged light to the total light ratio follows $\frac{\text{Tagged}}{\text{Total}}=\eta \frac{\text{Light passing through the ultrasound}}{\text{Total amount of light}}$, where η is the tagging efficiency. Here, we define the tagging efficiency η as the ratio between the power of light that is tagged to all the orders and the total power of light that passes through the ultrasound field, i.e., $\eta =\frac{P_{\text{tag}}}{P_{\text{untag}}+P_{\text{tag}}}$. We believe that η, rather than $\frac{\text{Tagged}}{\text{Total}}$, is the more appropriate definition for tagging efficiency because η directly relates to photons that actually passed through the ultrasound field and thus has an opportunity to interact with it. In comparison, $\frac{\text{Tagged}}{\text{Total}}$ relates to all photons including those that never passed through the ultrasound field and thus never has a chance to interact with it.

Light is scattered as it propagates through the scattering medium. This scattered light forms a speckle field. Here, we only consider light that passes through the ultrasound field. Consider the electric field E(t) of one speckle exiting the scattering medium. This speckle can consist of multiple photons, each of which can travel a different path through the medium. We have $E(t)=\underset{j}{\sum }E(s_{j},t)$, where $s_{j}$ is the trajectory of the j’th photon contributing to the speckle of interest. Assuming that different trajectories are independent, we can show that $E\{{\left|F[E(t)]\right|}^{2}\}=E\{\underset{j}{\sum }{\left|F[E(s_{j},t)]\right|}^{2}\}$ (see Sec. 7 Appendix), where E stands for expectation and ${\left|F[E(s_{j},t)]\right|}^{2}$ is the power spectrum. The tagging efficiency can be estimated from the ensemble average of the power spectrum of all the speckles. Therefore, we can estimate the tagging efficiency by sampling a subset of $s_{j}$ from all possible trajectories in the scattering medium.

The electric field of a given photon depends on the OPL. In order to compute the ${\mathrm{OPL}}_{j}(t)$, we need to first determine (1) the refractive index of the medium and (2) locations of all the particles along its trajectory j that caused the photon to get scattered. Given the acoustic pressure field at position r and time t as $P(r,t)=P_{0}\;\sin(\omega_{a}t-k_{a}\cdot r)$, where $P_{0}$ is the ultrasonic peak pressure, $\omega_{a}$ is the acoustic angular frequency, and $k_{a}$ is the acoustic wave vector. The refractive index change is $\Delta n=n_{0}(\frac{\partial n}{\partial p})P(r,t)$, where $n_{0}$ is the background refractive index without ultrasound, ∂n/∂p is the adiabatic piezo-optical coefficient of the medium. To determine the locations of all the particles, we model the particle displacement as A(r,t)=A0k^a sin(ωat−ka·r+ϕ), where $A_{0}$ is the amplitude of particle displacement, k^a is the unit vector of $k_{a}$, and ϕ is the phase difference between particle displacement and ultrasound pressure. We would like to determine $A_{0}$ and ϕ; to do so, we make the assumption that the particles move with the background fluid as it is compressed and rarified by the ultrasound.^25^ For simplicity, let us consider the case of a one-dimensional acoustic wave propagating along the direction y. Given the acoustic pressure field $P(y,t)=P_{0}\;\sin(\omega_{a}t-k_{a}y)$, the Euler equation can be written as $\rho \frac{\partial u}{\partial t}+\frac{\partial P}{\partial y}=0$, where u is the flow velocity and ρ is the density of the background medium. Notice that since $u=\frac{\partial A(y,t)}{\partial t}$, we have $\rho \frac{\partial^{2}A(y,t)}{\partial t^{2}}-k_{a}P_{0}\;\cos(\omega_{a}t-k_{a}y)=0$. This results in $A(y,t)=A_{0}\;\sin(\omega_{a}t-k_{a}y+3\pi /2)$, where $A_{0}=\frac{P_{0}}{\omega_{a}\rho v_{a}}$ and $v_{a}$ is the acoustic velocity. Note that when the particles move with the background fluid, there is a phase mismatch of ϕ=3π/2 between the particle displacement and the acoustic pressure field, or correspondingly the refractive index modulation. We explore the impact of this phase mismatch on tagging efficiency in simulation.

With the particle displacement expression in hand, we can compute the location of the m’th particle along trajectory j at time t as $r_{m}(t)=r_{0,m}+A(r_{0.m},t)$, where $r_{0,m}$ is the initial location of the m’th particle. Once we know the locations of the particles contributing to a given trajectory, as well the refractive index, we can then compute the OPL. The OPL is accumulated along trajectory j with all the free paths between scattering events, i.e., ${\mathrm{OPL}}_{j}(t)=\underset{m\in \text{trajectory }j}{\sum }{\mathrm{OPL}}_{j}^{m}(t)$. For the m’th free path at time t, the OPL is ${\mathrm{OPL}}_{j}^{m}(t)=\int_{0}^{s_{m}}n_{0}[1+\frac{\partial n}{\partial p}P_{0}\;\sin(\omega_{a}t-k_{a}\cdot r_{m})]dl_{m}$, where $r_{m}$ is the position along the m’th free path, $l_{m}$ is the distance along the m’th free path, and $s_{m}=|r_{m}-r_{m-1}|$ is the length of the m’th free path. Let k^m be the unit vector of the m’th free path, k^m=rm−rm−1sm, then rm=rm−1+k^mlm. Once we have OPL, we can then determine the electric field of a given photon. Then, we can compute the power spectrum, which gives light power at each frequency-shifted order. Finally, we average power spectrum for all the photons to get the estimation of tagging efficiency.

## 3 Simulation

**Fig. 2:**
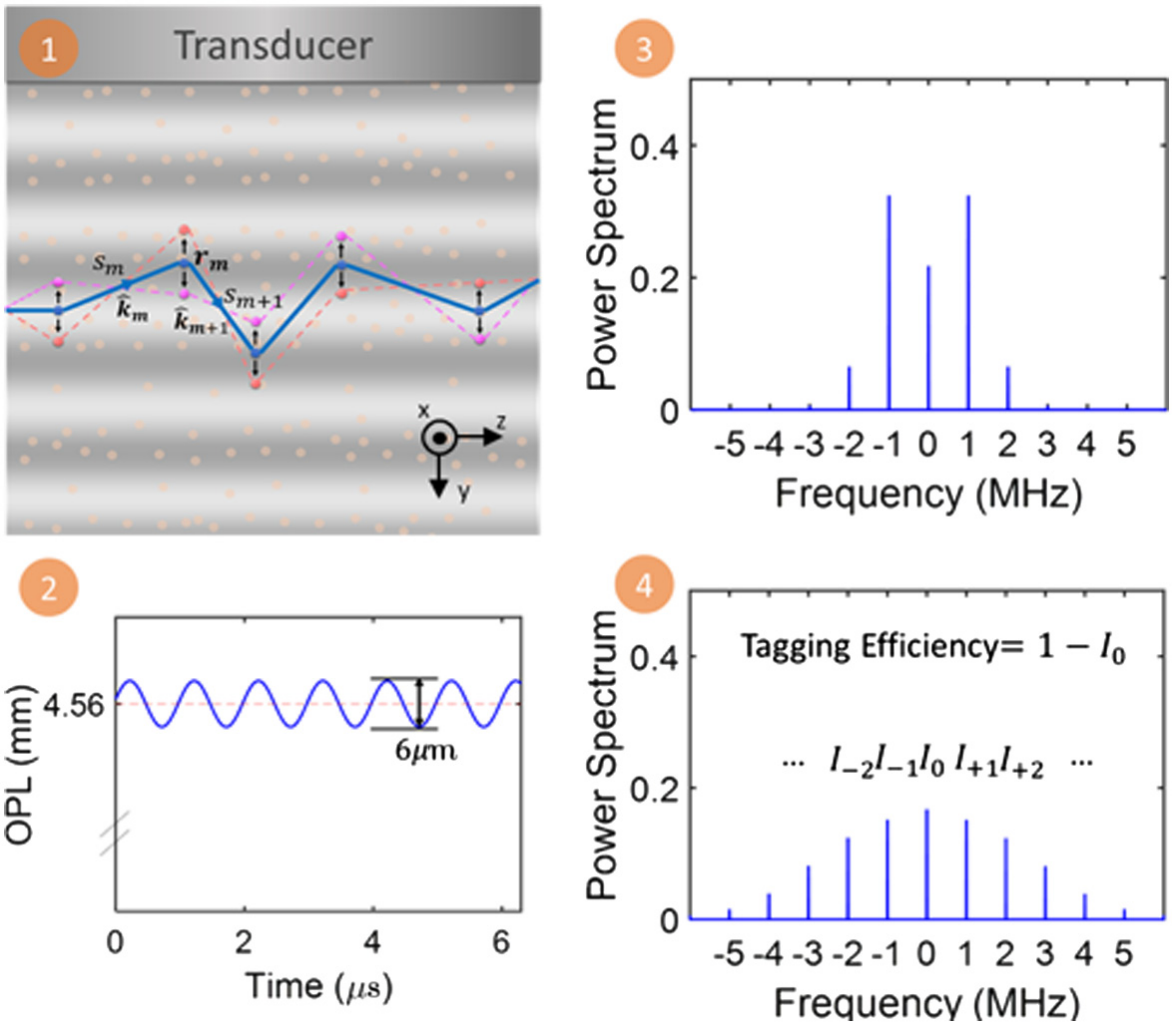
Flowchart of the simulation process. (1) Generate trajectories using Monte Carlo methods, (2) calculate OPL and the electric field considering refractive index changes and particle displacement comprehensively, (3) calculate power spectrum for each individual speckle, and (4) average to get the ensemble power spectrum and tagging efficiency.

As dictated by the theory, we simulated the ultrasonic modulation of the electric field and estimated the tagging efficiency from the ensemble power spectrum of all the trajectories. The simulation process is as follows (Fig. 2). (1) Using Monte Carlo methods, we generated the trajectories of the photon packets traveling in the medium for a given scattering coefficient $\mu_{s}$ and anisotropy g.^28^^,^^29^ (2) For each detected photon packet, we calculated the modulated OPL due to refractive index changes and particle displacement as ${\mathrm{OPL}}_{j}(t)$ and then the electric field for each photon packet as $E(s_{j},t)=e^{ik_{0}{\mathrm{OPL}}_{j}(t)}$, where $k_{0}$ is the optical wave vector in vacuum. (3) We calculated the power spectrum of each photon packet as ${\left|F[E(s_{j},t)]\right|}^{2}$. (4) Finally, we averaged the calculated power spectrum from each photon packet and normalized the ensemble power spectrum. The tagging efficiency was estimated as $1-P_{0}$, where $P_{0}$ is the zeroth-order power of the normalized ensemble power spectrum. In all our simulations, the acoustic pressure field was modeled to have a Gaussian distribution in order to match the pressure field of the ultrasound transducers used in the experiment. The light power distribution was uniform at the input surface in both our simulations and experiments.

**Fig. 3:**
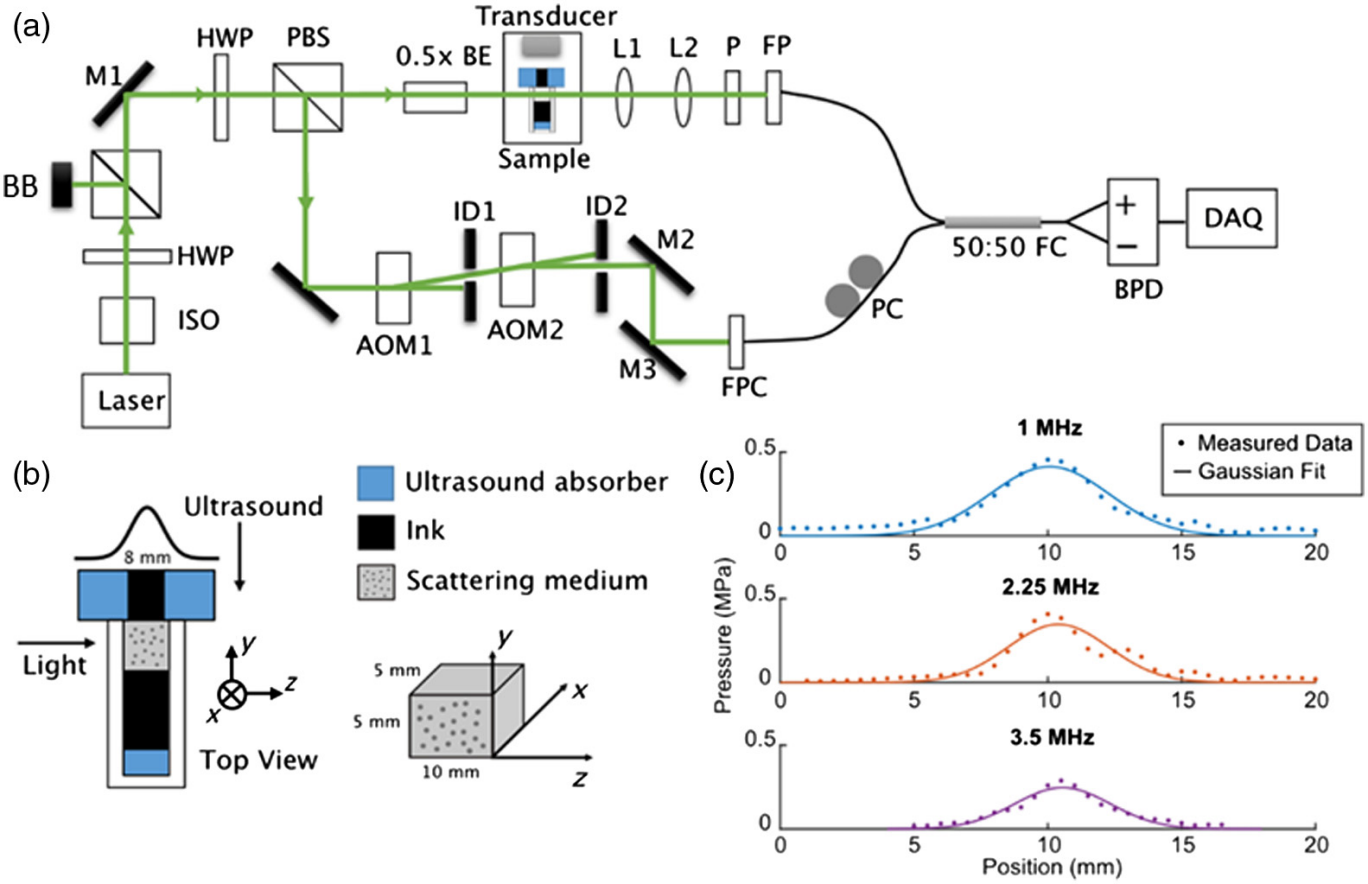
Diagram of the experimental setup. (a) The experimental setup consisted of a reference arm and a sample arm to detect light passing through the ultrasound field. ISO, isolator; HWP, half-wave plate; BB, beam block, M, mirror; PBS, polarized beam splitter; BE, beam expander; L, lens, AOM, acousto-optic modulator; ID, iris diaphragm; FPC, fiber port coupler; P, polarizer; FP, fiber port; PC, polarization controller; FC, fiber coupler; BPD, balanced photo-detector; DAQ, data acquisition card. (b) A zoomed-in view of the sample. (c). The ultrasound pressure field distribution of the three transducers used in the study.

The key part of simulation is the calculation of OPL. We describe the details here reusing the notations from Sec. 2. From Monte Carlo simulation, we obtain n trajectories passing through the ultrasound propagating volume. Each trajectory is stored by the locations of particles along the way. We treat all the trajectories as baseline trajectories. Assuming a continuous ultrasound wave with Gaussian lateral intensity distribution is propagating parallel to y axis and light is propagating along z axis before entering the scattering media [Figs. 3(b) and 3(c)]. For each of the trajectories, we compute its evolution at each time step by “moving” the particles along the trajectory according to A(y,t)=A0y^ sin(ωat−kay+ϕ). Notice that the phase mismatch ϕ comes into play when we calculate OPL. The time step is chosen to be small enough so that there would not be aliasing for the highest frequency of interest. To perform numerical integration for OPL, we discretize the volume into grids with size (Δx,Δy,Δz) and divide trajectories into pieces. Each piece is within one grid, and the length is denoted by $\ell_{i}$. The center of grid i is denoted by $r_{i}=(x_{i},y_{i},z_{i})$. Then, for each time step, we compute the refractive index in each grid by $n(r_{i},t)=\frac{\partial n}{\partial p}P_{0}e^{-\frac{{\left(x_{i}-x_{0}\right)}^{2}+{\left(z_{i}-z_{0}\right)}^{2}}{2\sigma^{2}}}\;\sin(\omega_{a}t-k_{a}y_{i})$, where $\left(x_{0},z_{0}\right)$ denotes the center of x–z plane and $\sigma^{2}$ is the variance of the Gaussian distribution. With the discretized trajectory and the refractive index, we compute OPL for baseline trajectory j at time t by summing up the piecewise OPL in all the grids, i.e., ${\mathrm{OPL}}_{j}(t)=\underset{i}{\sum }\ell_{i}n(r_{i},t)$.

**Fig. 4:**
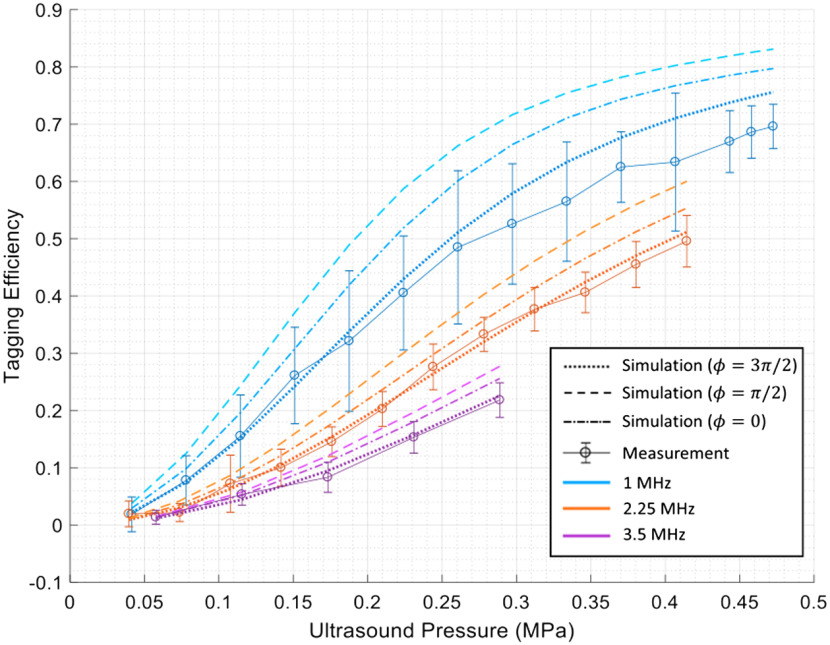
Tagging efficiency for 1, 2.25, and 3.5 MHz ultrasound versus ultrasound peak pressure. Dotted line shows simulation results and solid line shows experiment results. Each data point is an average tagging efficiency of 250 speckles. Error bar indicates the standard deviation of five measurements.

We also investigated the impact of phase mismatch between the acoustic pressure field P(r,t) and the particle displacement A(r,t) in simulation. Notice that the effect of the two mechanisms are coupled and should not be considered separately. We simulated the tagging efficiency for the case of ϕ=0, ϕ=π/2, and ϕ=3π/2 (the actual case). Comparisons are shown in Fig. 4.

## 4 Experimental Setup

The experimental setup is shown in Fig. 3(a). A laser with a wavelength of 532 nm and output power of 200 mW (Spectra-Physics, Excelsior-532-200-CDRH) was split into a reference and sample arms. The frequency of the reference arm, $f_{\mathrm{ref}}$, was set to be $f+f_{0}$, where f is the frequency of light, and $f_{0}$ was chosen to be lower than the acoustic frequency in order to separate the positive and negative orders in the power spectrum. In our setup, $f_{0}$ was selected to be 50 kHz. This is achieved by first upshifting the light frequency by 80.05 MHz using an acousto-optic modulator (AOM1) and then downshifting it by 80 MHz using a second AOM (AOM2). After shifting, the reference beam was coupled into one arm of a 2×2 fiber coupler (Thorlabs, TN532R5A2). A polarization controller (PC) was used to match the polarization state of the reference arm and sample arm.

For the sample arm, the sample beam was first demagnified by 2× and then transmitted through the sample. The speckles on the back plane of the sample were relayed by a 4f system to the other arm of the 2×2 fiber coupler. The sample arm optics was designed such that the light power coming from only a single speckle was detected at a given time. A polarizer was also added to ensure that the light was linearly polarized to maximize the strength of the interference signal. A balanced photodetector (BPD) (Thorlabs, PDB440A) was used to measure the interference signal.

The interference signal detected on positive and negative monitor arms of the BPD can be mathematically expressed as

$$P_{+}(t)={\left|E_{\text{untag}}e^{i2\pi ft}+E_{\text{tag},n}e^{i2\pi (f\pm nf_{\text{us}})t}+E_{\mathrm{ref}}e^{i2\pi (f+f_{0})t}\right|}^{2}\;={\left|E_{\text{untag}}\right|}^{2}+{\left|E_{\text{tag}}\right|}^{2}+{\left|E_{\mathrm{ref}}\right|}^{2}+2E_{\mathrm{ref}}\overset{\infty }{\underset{n=-\infty }{\sum }}E_{\text{tag},n}\;\cos[2\pi (nf_{\text{us}}\pm f_{0})t]\;+2E_{\mathrm{ref}}E_{\text{untag}}\;\cos(2\pi f_{0}t)+2E_{\text{untag}}\overset{\infty }{\underset{n=-\infty }{\sum }}E_{\text{tag},n}\;\cos(2\pi nf_{\text{us}}t)$$

$$P_{-}(t)={\left|E_{\text{untag}}e^{i2\pi ft}+E_{\text{tag},n}e^{i2\pi (f\pm nf_{\text{us}})t}-E_{\mathrm{ref}}e^{i2\pi (f+f_{0})t}\right|}^{2}\;={\left|E_{\text{untag}}\right|}^{2}+{\left|E_{\text{tag}}\right|}^{2}+{\left|E_{\mathrm{ref}}\right|}^{2}-2E_{\mathrm{ref}}\overset{\infty }{\underset{n=-\infty }{\sum }}E_{\text{tag},n}\;\cos[2\pi (nf_{\text{us}}\pm f_{0})t]-2E_{\mathrm{ref}}E_{\text{untag}}\;\cos(2\pi f_{0}t)+2E_{\text{untag}}\overset{\infty }{\underset{n=-\infty }{\sum }}E_{\text{tag},n}\;\cos(2\pi nf_{\text{us}}t).$$

The output of the BPD, which is the difference between the positive and negative ports, was read out by a 16-bit data-acquisition card (GaGe, CSE1622, 16 bit, 200  MS/s). The benefits of using a BPD are (a) to reduce the laser fluctuation noise from the common term in $P_{+}$ and $P_{-}$ and (b) to reduce the beating signal between untagged light and tagged light. Setting the frequency of the reference arm to be $f+f_{0}$ enabled us to distinguish both the positive and negative orders of the tagged light on the power spectrum. Specifically, untagged light will appear as a signal at frequency $f_{0}$ and ±n’th order of tagged light will appear at $f=nf_{\text{us}}\pm f_{0}$.

At the output of the BPD, we get $P(t)=P_{+}(t)-P_{-}(t)$. We did the measurement when ultrasound is on and off, respectively, to get $P_{\text{on}}(t)$ and $P_{\text{off}}(t)$. Then, we calculated the power spectrum ${\left|F[P(t)]\right|}^{2}$ of the signal and normalized it by dividing the sum of the power spectrum. The zeroth-order of light corresponds to the untagged light. When ultrasound is off, there is only the zeroth-order in the power spectrum. Let $P_{0,\text{on}}$ and $P_{0,\text{off}}$ be the relative power of zeroth-order light from the normalized power spectrum when ultrasound is on and off, respectively. Then, the tagging efficiency was calculated by $\frac{P_{0,\text{off}}-P_{0,\text{on}}}{P_{0,\text{off}}}$.

We measured the tagging efficiency at three different ultrasound frequencies: 1 MHz (Olympus, U8517028), 2.25 MHz (Olympus, U8422015), and 3.5 MHz (Olympus, U8422021). For each transducer, we measured the tagging efficiency at various pressure values. To measure the tagging efficiency, we first scanned the fiber port to measure the light power variation over time for 250 different speckles. Then, we calculated the power spectrum for each speckle and took the ensemble average over the 250 different measurements to get an estimate of the tagging efficiency. For each pressure value, the measurement was repeated five times, and the mean and standard deviation were recorded.

The scattering sample was made from 0.75-μm-diameter polystyrene beads (Polybead Microspheres, No. 07309-15, Polysciences) embedded in a gel made from deionized water with carrageenan (No. C1013-100G, Sigma-Aldrich). The radius and concentration of polystyrene beads (1.5  g/mL) were chosen to achieve a scattering anisotropy g=0.9 and a scattering coefficient $\mu_{s}=4\;{\mathrm{mm}}^{-1}$. The scattering sample was placed in a sample holder, which was then immersed in a water tank. To only measure the tagging efficiency of light in the scattering medium, we needed to make sure that there is no ultrasound–light interaction in the transparent region because otherwise Raman–Nath diffraction will contribute to the tagging efficiency. The transparent regions are the water surrounding the sample holder and the gel surrounding the scattering medium. To avoid ultrasound–light interaction in water, we added a single slit made from ultrasound absorber in front of the sample to confine the ultrasound field to the scattering medium, as shown in Fig. 3(b). We also added ink to the transparent gel within the sample holder to absorb the light. Therefore, only the photons that passed through the ultrasound field were detected. We also placed ultrasound absorber at the bottom of the sample holder to prevent ultrasound echo. The ultrasound pressure at 5 mm distance behind the slit was measured using a hydrophone (ONDA HNR-0500 hydrophone), and the profiles are shown in Fig. 3(c) for unfocused transducers of different frequencies. As we can see, the ultrasound lateral profile approximately follows a Gaussian distribution.

## 5 Results

Figure 4 shows the measured tagging efficiency as a function of ultrasound pressure for three different ultrasound frequencies: 1 MHz (in blue), 2.25 MHz (in red), and 3.5 MHz (in purple). The range of ultrasonic pressure was different for the different transducers, as it depends on the maximum power the transducer could endure. From the experimental results (in solid lines), we see that increasing power corresponds to higher tagging efficiency. For the 1 MHz ultrasound, the tagging efficiency was about 70% when the ultrasound pressure was about 0.47 MPa. Moreover, for the same ultrasound pressure, higher frequency corresponded to lower tagging efficiency. This is because particle displacement is inversely proportional to the ultrasound frequency. Thus, higher frequency ultrasounds generate smaller particle displacements for the same pressure, resulting in lower tagging efficiency.

Figure 4 also compares the simulation results for ϕ=3π/2 (dotted lines) to the experimental results (solid lines). The experimental and simulation results correspond well. To investigate the impact of the phase mismatch on tagging efficiency, we also simulated and plotted the tagging efficiency for the case of ϕ=0 (dotted dashed lines) and ϕ=π/2 (dashed lines). We also simulated the case when ϕ=π and the tagging efficiency is similar to the case when ϕ=0 and thus is omitted in Fig. 4 for clarity of presentation. Comparing the results with different phase mismatches, we observe that the phase mismatch results in different tagging efficiency. When ϕ=3π/2 (the actual case), the simulated tagging efficiency is the lowest and corresponds well with the experimental results. When ϕ=π/2, the simulated tagging efficiency is much higher than the measured tagging efficiency. When there is no phase mismatch, the simulated tagging efficiency lies in between. That implies that when ϕ=3π/2, the particle displacement modulation cancels out part of the refractive index modulation in our simulation. Intuitively, when the particle moves along with the background medium, the OPL changed by particle locations cancel out part of that changed by refractive index.

**Fig. 5:**
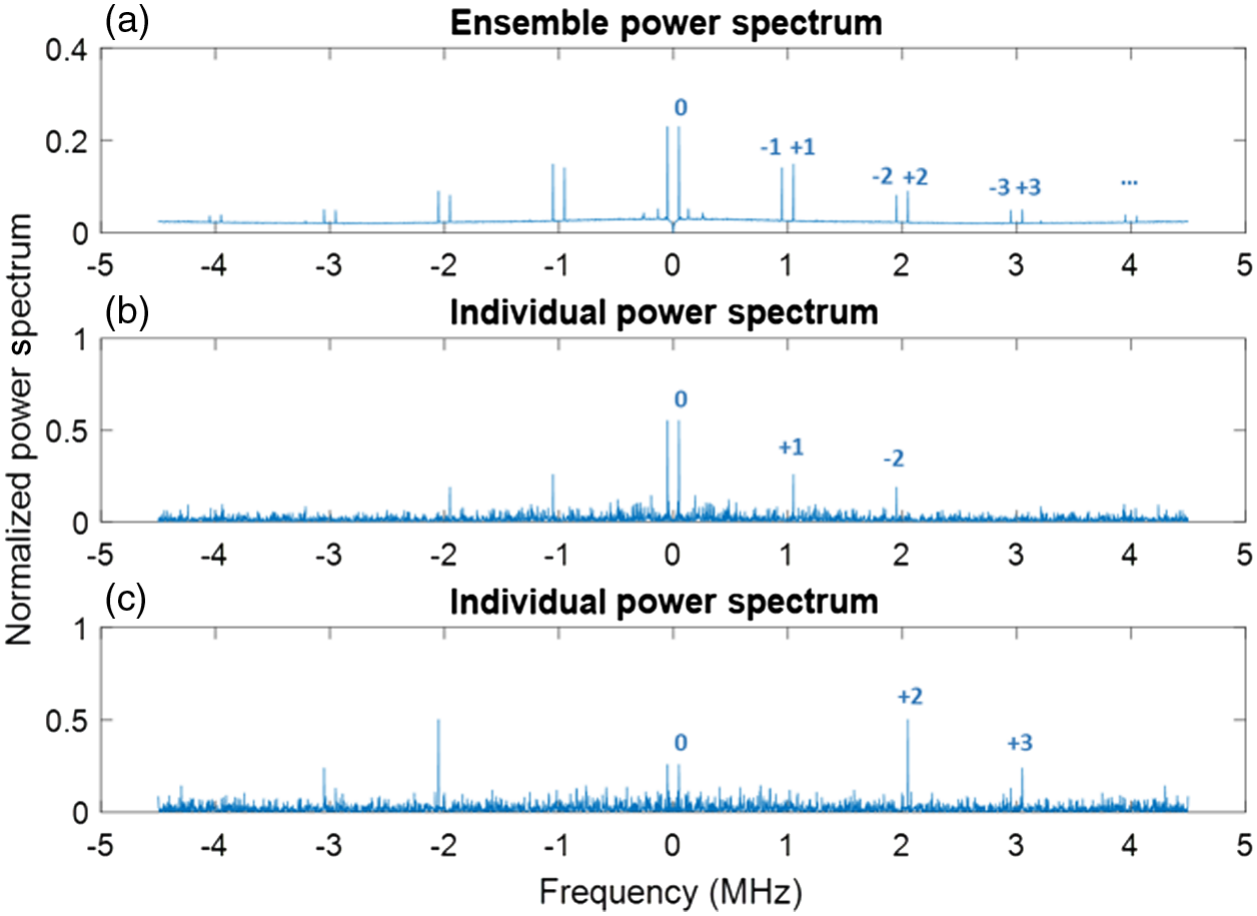
Normalized power spectrum of light passing though 1-MHz ultrasound field in scattering medium. The blue numbers in the plot indicate the order of the peak (with a frequency of $nf_{\text{us}}\pm f_{0}$). (a) Ensemble power spectrum of 1250 speckles. (b) Power spectrum of one speckle arbitrarily chosen from the speckle field. (c) Power spectrum of another speckle arbitrarily chosen from the speckle field.

To see the power of light at each order, we also investigated the power spectrum of the scattered light passing through the ultrasound field. For the 1-MHz ultrasound at 0.47 MPa, Fig. 5(a) shows the ensemble power spectrum of 1250 speckles. Figures 5(b) and 5(c) show the power spectra of two different speckles arbitrarily chosen from the speckle field. As expected, the strength for the positive and the negative orders is equal in the ensemble power spectrum, i.e., the probability of a photon to be upshifted or downshifted equals to each other. However, this is not true for individual speckles. As shown in Figs. 5(b) and 5(c), light power at the positive orders and the negative orders is asymmetric for an individual speckle. In Fig. 5(b), the tagged light has frequency of $f_{0}+f_{\text{us}}$ and $f_{0}-2f_{\text{us}}$, whereas in Fig. 5(c), the tagged light has frequency of $f_{0}+2f_{\text{us}}$ and $f_{0}+3f_{\text{us}}$. This is because light at different frequencies has different speckle field after passing through the ultrasound field.

## 6 Discussion and Conclusion

The main challenge for UOT and other ultrasound-assisted optical techniques is to isolate the small signal of tagged photons from a large background of untagged light. Since the detection methods usually detect only the first order of the tagged light, tagging efficiency typically refers to the ratio between the power of the light at the first order and the total power. It is conventionally believed that the tagging efficiency is low (∼10% or less as a rule of thumb). However, it is important to note that light is modulated to multiple orders, and the tagging efficiency accounting for all of the frequency shifted light could be high. In addition, the light that does not pass through the ultrasonic region should not be considered into calculating tagging efficiency since the light diffusive region could be arbitrarily large. The definition of tagging efficiency as the power of all frequency-shifted light over the power of the light that passes through the ultrasound region, which could also be computed as (1, the fraction of unmodulated light), is more appropriate and consistent with the term. We showed that if we adhere to the second definition of tagging efficiency, we can experimentally measure tagging efficiency to be ∼70% under certain circumstances.

This surprisingly high tagging efficiency is multiple folds higher than the <10% tagging efficiency associated with first-order modulation. These findings provide us with insights in designing UOT-related experiments. Although the power of the first order of the tagged light might be small, the power of tagged light at all the orders could be larger. If we detect all the tagged light with different frequencies, we can have a significantly improved system SNR.

On a different topic, our work showed that the two ultrasound modulation mechanisms actually counteract each other in a scattering medium. The OPL modulation from refractive index change will be partly “undone” by the particle movement because the particles in the scattering medium move with the background medium when ultrasound propagates. Under this movement assumption, we showed that the phase mismatch between the pressure field and particle displacement is 3π/2, which is used in our simulation to get the predicted tagging efficiency. This prediction is well supported by our experimental results as our simulation results associated with the 3π/2 phase mismatch match well with our experimental measurements. In contrast, simulation results with 0 and π/2 phase mismatch are ill matched with the experimental results.

By recording a time series of the interference signal, our system is able to detect the power spectrum of light without sweeping the frequency of reference beam, which provides us a more comprehensive understanding of ultrasound tagging in scattering medium. By choosing the reference beam frequency to be untagged light frequency plus some low frequency, i.e., $f_{\mathrm{ref}}=f+f_{0}$, we can separate the light at the positive and the negative orders. In general, ± orders of light partially destructively interfere because of phase mismatch. If the reference beam frequency is set to be the frequency of untagged light, i.e., $f_{0}=0$, then light at ± orders will coalesce at frequency of $nf_{\text{us}}$ and partly cancel out. Researchers have used cameras for parallel detection of ultrasound tagged light.^30^^,^^31^ Due to the speed limit of the camera, the frequency of the reference beam has to be adjusted to detect each frequency order.

From the normalized power spectrum, we noticed the following: (1) light power at higher order frequencies is nonnegligible, as shown in Fig. 5(a). In particular, the power ratios between tagged light at ±1, ±2, and ±3 order and total amount of light are ∼29%, ∼17%, ∼10%, respectively. (2) Individual speckles have different power distributions at different frequency orders as Figs. 5(b) and 5(c) show, which makes sense because light at different frequencies will have different speckle patterns after passing through the scattering medium.

Our experimental results also show that tagging efficiency is higher when the ultrasound frequency is lower under the same ultrasound pressure, which means that more photons are tagged and better SNR can be achieved with low-frequency transducers. However, in UOT or TRUE experiments with focused ultrasound, high-frequency ultrasound provides smaller focal spot sizes, resulting in higher resolution. Therefore, researchers should balance the resolution and SNR requirements to choose appropriate transducers.

In conclusion, we quantitatively studied ultrasound–light interaction in scattering media. We simulated and experimentally measured ultrasound tagging efficiency as a function of ultrasound frequency and pressure. Our system is able to measure the power spectrum of the light passing through the ultrasound field, which gives more insights in tackling the SNR issue in ultrasound-assisted optical experiments.

## 7 Appendix

Proof of $E\{{\left|F[E(t)]\right|}^{2}\}=E\{\underset{j}{\sum }{\left|F[E(s_{j},t)]\right|}^{2}\}$:

The electrical field at one point can be written as the summation of electric field from all the trajectories that end at that point and so as its Fourier transform:

$$E(t)=\underset{j}{\sum }E(s_{j},t)\;F[E(t)]=\sum_{j}F[E(s_{j},t)].$$

Denote order i of $F[E(s_{j},t)]$ as ${\left\{F[E(s_{j},t)]\right\}}_{i}=A_{j}e^{i\phi_{j}}$. Then order i of F[E(t)] is

$$F{\left[E(t)\right]}_{i}=\underset{j}{\sum }{\left\{F[E(s_{j},t)]\right\}}_{i}=\underset{j}{\sum }A_{j}e^{i\phi_{j}}.$$

Therefore, we have

$$E({\left|\underset{j}{\sum }A_{j}e^{i\phi_{j}}\right|}^{2})=E(\underset{j}{\sum }A_{j}^{2}+\underset{i\neq j}{\sum }A_{i}A_{j}\;\cos\;\phi_{i}\;\cos\;\phi_{j}+\underset{i\neq j}{\sum }A_{i}A_{j}\;\sin\;\phi_{i}\;\sin\;\phi_{j})\;=E(\underset{j}{\sum }A_{j}^{2}).$$
